# Diagnostic X‐ray facilities as per quality control performances in Tanzania

**DOI:** 10.1120/jacmp.v7i4.2291

**Published:** 2006-11-28

**Authors:** Yesaya Y. Sungita, Simon S.L. Mdoe, Peter Msaki

**Affiliations:** ^1^ Tanzania Atomic Energy Commission P.O. Box 743 Arusha Tanzania; ^2^ Physics Department University of Dar Es Salaam P.O. Box 35063 Dar Es Salaam Tanzania

**Keywords:** quality control, repair, preventative maintenance

## Abstract

Without appropriate quality control (QC) and preventative maintenance (PM) measures for X‐ray machines in place, the benefits of reduced dose to the patient and early diagnosis will not be realized. Quality control and PM also make it possible to unify X‐ray‐imaging practices in the country using international image quality guidelines. The impetus for the present work resulted from the concern that with the recent increase in the numbers of X‐ray machines in Tanzania, but with limited technical support to maintain and operate them, can increase radiation risk to patients and lower diagnostic accuracy. The aim of this work is to report on the current status of diagnostic X‐ray machines in Tanzania in order to produce the data needed to formulate QC and PM policies and strategies. These policies and strategies are needed to ensure that patients receive the lowest possible radiation risk and maximum health benefits from X‐ray examinations. Four QC tests were performed on a total of 196 X‐ray units. Accurate beam alignment and collimation were tested on 80 (41%) units, the timer accuracy was tested on 120 (61%) units, and a radiation leakage test was performed on 47(24%) units. Preventative maintenance tests were performed on all 196 X‐ray units. The results showed that of the units tested for QC, 59% failed the kilovoltage (kVp) test, 57% failed the timer accuracy test, 60% failed the beam alignment test, and 20% failed the radiation leakage test. Only 13% of the units passed the PM test: 53% of the units were defective, and 34% were out of order. As a result of the PM findings, the government has introduced a rehabilitation project to service X‐ray units and replace nonoperational X‐ray units. The new units have full support service contracts signed by their suppliers. As a result of the QC findings, X‐ray maintenance retraining programs have been introduced.

PACS numbers: 87.52.‐g, 87.52.Tr, 87.58.SP, 87.59.Bh

## I. INTRODUCTION

The need for frequent quality control (QC), preventative maintenance (PM), and repairs (in case of total breakdown) are well known: lack of such services leads to underutilization of expensive medical equipment, thereby making health‐care services in any country less cost‐effective. The problems of QC and PM in Tanzania are on the increase; this is the case because the demand for modern medical equipment in the health‐care sector has increased in recent years, but this has not been accompanied by a commensurable increase in qualified personnel in medical electronics to provide the needed services. Using service engineers from foreign suppliers to overcome this problem is not a viable option; this is because the service is not available under short notice, and when available it is prohibitively expensive. Therefore, local medical centers in Tanzania have continued to rely on the scarce services provided by the qualified local personnel in medical electronics. Maintenance and repair of medical equipment in Tanzania are further worsened by the general lack of service manuals, spare parts, and suitable environmental conditions in which to operate the equipment once repaired.^(^ ^1^ ^,^ ^2^ ^)^ This, coupled with the chronic shortage of personnel in medical electronics and financial resources, has exacerbated the problem by lowering the possibilities for followup actions based on the results of QC procedures.

The benefits of using high‐quality X‐ray images in diagnosing disease and in the guidance of therapeutic procedures are well known. X‐rays are used in the diagnosis of many diseases and disorders, and they help clinicians confirm or rule out a diagnosis. The risk to individuals from the radiation used in diagnostic X‐rays is small compared to the benefits that accurate diagnosis and treatment can provide. Unfortunately, due to the rapid increase in the number of X‐ray units in Tanzania, many users of X‐ray equipment do not understand the basic principles of radiation protection, thus maximizing the associated radiation risks to patients. The benefits of diagnostic X‐rays are drastically reduced when the equipment is operated without adequate QC and maintenance. In most cases, such equipment has higher radiation risks because of an increased dose in a single exposure and repeated exposure attributed to low image quality.^(^ ^3^ ^,^ ^4^ ^)^ Quality control is also important in nations that use or intend to use objective assessment of radiological images and international image quality guidelines to unify X‐ray‐imaging practices nationally and internationally.^(^ ^5^ ^,^ ^6^ ^)^ The aim of this work is to report on the current status of diagnostic X‐ray machines in Tanzania in order to produce the data required to formulate and implement QC and PM policies and strategies. The impetus for this program came as a result of the concern that the recent increase in the acquisition of X‐ray machines in Tanzania with limited technical support to maintain and operate such equipment can severely undermine the anticipated health benefits from diagnostic X‐ray examinations.

## II. MATERIALS AND METHODS

### A. Centers with diagnostic X‐ray units

The 196 diagnostic X‐ray units studied are located in 19 regions in Tanzania. Four QC tests were performed: accurate beam alignment and collimation were tested on 80 (41%) units, kilovoltage (kVp) accuracy and reproducibility were tested on 120 (61%) units, the timer accuracy was tested on 120 (61%) units, and a radiation leakage test was performed on 47 (24%) units. Preventative maintenance tests were performed on all 196 X‐ray units. Detailed descriptions of each test are given separately below.

### B. Beam alignment and collimation

**Figure 1:**
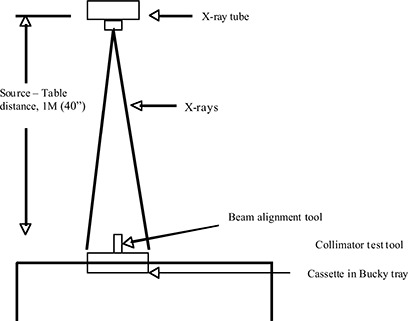
Placement of collimation and beam alignment test tools for proper exposure

Collimation/image receptor alignment checks and ascertains the adequacy of cones, congruence of light and radiation field, and accuracy of collimator X‐ray scales and tracking or automatic collimator with cassette size. Proper alignment of the X‐ray beam and the image receptor leads to centering of images on the area of interest, reduction of scatter radiation with a consequent increase in contrast, and reduced gonadal exposure; in addition, it also prevents irradiation of areas not required in the images and reduces repeated examination. The beam alignment and collimation compliance tests were performed using the Radiological Medical Imaging (RMI) quality assurance handbook and the ICRP recommendations.^(^ ^7^ ^,^ ^8^ ^)^ The experimental setup for the tests is shown in Fig. 1. The beam alignment and collimator test tools were placed on the examination table with the radiographic cassette in the bucky tray. The distance between the X‐ray tube and the top of the examination table was set at 1 m (source‐to‐detector distance). The X‐ray tube directed the central ray downward. The collimator shutters at the X‐ray tube were then adjusted in such a way that the edges of the light field coincided with the rectangular outline on the collimator test tool. The radiographs of the beam alignment and collimator test tools were done on the 8×10 cassette. The exposure parameters to give good radiographs were selected according to the power rating of the machine. In most cases, the exposure parameters used were 60 kV, 0.05 s, and 20 mA for portable X‐ray machines.

Good collimation was assumed if the X‐ray field fell just within the image of the rectangular frame in test tools. For example, if the edge of the X‐ray field fell on the first spot, +1 cm on either side of the line, the X‐ray and light field was misaligned by 1% of the distance between the X‐ray source and the table. In this compliance test, the maximum allowable misalignment was 2% of the source‐to‐image distance. The X‐ray beam alignment was considered proper if the images of the two steel balls on the test tool were perpendicular to the central ray, or within 1.5±. The X‐ray beam was set such that it was perpendicular to the image receptor on the plane of the table (Fig. 1).

### C. Kilovoltage tests

Kilovoltage is an important parameter to consider when choosing radiographic technique factors. The strength and, hence, the penetration power of the X‐rays are determined by the kV settings. Generator control and kVp calibration determine accuracy and consistency of X‐ray production. The kVp tests were done as indicated in the RMI quality assurance handbook. The instrument used was an RMI digital kVp meter, model 230. The exposure parameters were selected depending on whether the equipment was a single‐ or three‐phase unit. For a single‐phase unit, the time selected was >0.2 s, mA>20 mA per radiograph, and for the three‐phase units, the time set was >0.1 s. Three different kVp settings were tested and data collected three times to ascertain reproducibility. The QC procedures require that the X‐ray tube voltage variation be within ±4 kVp or ±5% of the normal value, whichever is less, within 150 ms of initiating the exposure, assuming that the equipment is operating in a stable manner. However, for the sake of radiation protection safety standards, a variation of up to ±10% was tolerated.

### D. Timer test

The uniform intensity of X‐rays is determined by invariable tube current and exposure time during X‐ray production. The uniform intensity of X‐rays enables the same exposure parameters to give similar quality radiographs. The reproducibility of timer accuracy and mA calibration tests are important parameters to ascertain X‐ray production quality. Using a digital X‐ray exposure timer (RMI, model 221A), we tested the timer accuracies of X‐ray exposures. The source‐to‐detector distance was set at 100 cm for the three‐phase units and 75 cm for the single‐phase unit. The adjustment of a beam‐limiting device to limit an X‐ray field at the detector to about 2.5 cm squares was done. The X‐ray exposures were made on the detector for three timer settings. In most cases, the exposure technique factors used were 80 kV and 200 mA. The equipment passed the QC tests if the variation in the timer values was within 5% or 2 ms, whichever error was larger.

### E. Tube leakage test

The leakage measurements for the X‐ray tube were done at 100 cm, focus‐to‐detector distance positions at four different sides of the tube. The exposures were made with the collimator shutters at X‐ray fully closed and X‐ray tube projected vertically downward. The exposure parameters for the leakage tests were 125 kV and 250 mAs for most units (Bethold dosimeter model LB 1310, with X/T probe model KZ25P). However, in some cases, it was not possible to perform all of these procedures because either the films, the film processing chemicals, or both were lacking. Some units did not meet the required specifications for certain QC procedures.^(^ ^9^ ^–^ ^10^ ^)^ Also, unavailability of some test monitoring instruments during survey work prevented some QC procedures from being completed.

## III. RESULTS

Most of the X‐ray equipment installed in the country is Philips and Siemens, as shown in Table 1. The equipment test were all those installed in center before the year 2000 (Table 2).

**Table 1:** Distribution of brands of radiological installations

Brand	Good working order	With defect	Out of order	Number of machines
Philips	12	49	31	92 (46.9%)
Siemens	8	36	20	64 (32.7%)
General Electric	0	1	4	5 (2.6%)
Todd Research	1	3	1	5 (2.6%)
Picker	0	3	3	6 (3.0%)
Others	4	12	8	24 (12.2%)
Total (%)	25 (12.8%)	104 (53%)	67 (34.2%)	196 (100%)

**Table 2:** Distribution of year when some of the radiological units surveyed were put into service (1957–2000)

Period	1957–1960	1961–1970	1971–1980	1981–1990	1991–2000
Number of machines	2	22	32	26	30

The results of QC tests for X‐ray units are shown in Table 3. Equipment tested for kVp calibration showed unacceptable errors ranging from 4 kVp to 20 kVp.

**Table 3:** Results of quality control performance for some of the X‐ray units

Quality control test	Units tested	Pass quality control tests		Units not tested
kilovoltage (accuracy and reproducibility)	120 (61.2%)	49 (41%)	71 (59%)	76 (39.2%)
Timer accuracy	120 (61.2%)	69 (57.5%)	51 (42.5%)	76 (39.2%)
beam alignment and collimation	80 (40.8%)	48 (60%)	32 (40%)	116 (59.2%)
leakage tests	47 (24%)	39 (19.9%)	8 (4.1%)	149 (76%)

When the X‐ray machines were tested for timer accuracy, a typical example of the problems seen was this: the timer setting was 1.00 s, and the recorded time was 1.689 s, with an error of 0.689 s, a very serious problem. Some X‐ray equipment operators have been forced to estimate or guess exposure times during due to defects in the timer circuit.

Misalignment of the light and the X‐ray field for most X‐ray units tested indicated that the X‐ray and light fields were misaligned by more than 2% (±2 cm), which is unacceptable. The beam in some units also showed that the central ray was more than 3± from the perpendicular, although few units were misaligned to that extent. However, in most cases, the maintenance personnel of the research team rectified the problems. Most of the X‐ray machines tested for tube leakage gave results that were below 0.5 mSv/h at 1 m, which complied with safety requirements.

## IV. DISCUSSION

During the survey, there were various problems identified that contribute to the breakdown, multifunction, and consequent poor QC performance of most equipment. These problems are divided into three main categories: procurement and acceptance problems, maintenance and repair problems, and common causes of failure rate.

### A. Procurement and acceptance problems

The survey revealed that some equipment had been damaged due to environmental conditions while in storage. Either the center was not ready for the immediate installation upon delivery of the equipment, or there was no qualified installation personnel. At some hospitals, equipment has been waiting for installation for up to four years after delivery. The failure to install the equipment was caused by delays in finishing the construction of the X‐ray building, or some important items for installation were not ready at the site. Some X‐ray buildings had the electrical installations supplied with a single‐phase line while the equipment to be installed was rated for three‐phase lines. Efforts to lay the three‐phase line had taken so long that the machines were on the verge of being damaged due to poor storage. Some hospitals received donated X‐ray equipment with 110 AC power rating with line frequency of 60 Hz, which needed some adaptation of the main transformer and timing circuits to 230 AC, 50 Hz. In some cases, the buildings intended for the X‐ray rooms were being used for other activities, which indicated a change in priority. To emphasize, the consideration of lead time before and upon delivery of equipment with necessary preparations should be done for acceptance and essential installation of equipment. After delivery, the equipment should be inspected to evaluate if it meets electrical and QC specifications as specified from the purchase orders.^(^ ^9^ ^,^ ^12^ ^)^ The source of funding for the equipment should be well planned, whether it is from the institutional budget, grant, or donation from another organization. The relevant authority based on legal framework of the institution should approve the procurement. The insurance and packaging, after sales services, a training component, and an import and acquisition license, if any, should be considered to ensure the safe delivery and reliable operation of the equipment as expected.^(9)^

### B. Maintenance and repair problems

It was observed during the survey that most of the operation and circuit manuals had been misplaced, lost, not supplied, or taken away by service technicians during repair missions. With regard to maintenance records, it was also noted that most of the equipment had no maintenance records. Repairs had been done, and the users were left without any records to show what had gone wrong and what repairs had been carried out or if there had been any circuit adaptation or refurbishment. Unavailability of spare parts and prohibitive financial constraints account for poor quality repair services.

### C. Common cause of failure rate

Most of the X‐ray machines, particularly the Siemens and Watson models, were observed to be more than 20 years old, as shown in Table 3. We could not obtain installation records of all the machines due to old age and inadequate record keeping.

In many cases, the Siemens Nanophos X‐ray machines had a defective main switch for compensation. These switches had worn out due to old age. The kVp selection switches, particularly the switch contact plates, were common defective parts. The timer problems have also been recorded to be among the most defective parts of X‐ray units. Erroneous timing reading might be caused by waveform problems, such as pulses of different height, too low radiation intensity or low‐peak intensity at the beginning of the exposure, and a faulty timing circuit. A defective X‐ray tube was also recorded as a common problem. Most of the fluoroscope options on the Siemens models are defective.

The common faults with Philips X‐ray machines were in tube heads, light beam diaphragms, and exposure controls. There has also been a wear and tear of mechanical parts as a result of careless treatment of all moving parts, bucky trays, and light beam diaphragms. For mobile units in particular, movement has usually been without proper care, especially up and down slopes, sometime causing trapping and overriding the cable and the consequences of bad handling.

The poor quality of electrical power is another major cause of equipment malfunction or breakdown. Only five pieces of equipment were recorded to have line conditioning (i.e., voltage stabilization) and installed in air‐conditioned rooms; the rest were not, despite the high temperatures and humidity in this tropical country. Some X‐ray rooms have a leaky roof and open windows that allow rain spills to enter the room.

At the time of the survey it was observed that due to the problems pointed out earlier, there was no followup action on the QC results such as making the necessary corrective maintenance. There was also a problem of coordination/feedback between personnel who perform QC procedures and service agents who were supposed to perform the corrective maintenance. It should be noted that QC, PM, and repairs are integral procedures in that way complementary to radiation protection procedures.

## V. CONCLUSIONS AND RECOMMENDATIONS

Detailed information on the status of equipment surveyed has been collected, and a database to that effect has been prepared and is available. The results on analysis of the foregoing situation showed that about 12% of equipment passed the quality control tests. Thirty‐four percent of the equipment surveyed was found to be completely out of order. The remaining 54% of the equipment was working with some defects, and some equipment had poor QC results.

It is intended that the results of this survey help in establishing a planned national preventative maintenance program to protect investment in the equipment, through regular and adequate maintenance.

In order to avoid wasting resources due to overlapping and duplication of QC and PM efforts, a coordinated approach through external collaboration is a prerequisite to the implementation of a national QC and PM program. Medical equipment is owned by different institutions: the government, religious and charity organizations, companies, and private institutions. Each may have a different approach toward implementation of QC an PM programs, taking into account the restraints imposed by socioeconomic factors, such as location, the environment, funding, the level of medical expertise, and available technical know‐how. Due considerations must therefore take into account this diversity in developing the necessary skills and coordination mechanisms. Based on the realistic appraisal of maintenance costs and available financial resources, a maintenance program should include both in‐house and contracted services. It is realistic to use the latter where available and use the in‐house service facilities for less accessible centers in the region or zone. The aim there should not be to compete with outside service agents, but rather to collaborate with them in order to make the two complementary.

Since preventative maintenance, repair, and quality control require availability and the proper balance of various levels of skills, it is recommended that the integrated approach to the program should start with the users who have the basic knowledge of PM, QC, and fault detection. These levels should include in‐house technicians capable of performing minor repairs. The second level should require personnel with a higher level of skill and a higher technical education. Engineering departments of consultant hospitals or regional hospitals may provide this. They should offer services to the zone in which the workshop is situated. The third level requires well‐trained technicians possibly with factory training for various pieces of equipment. The services of this level are supposed to be provided by the central government laboratories, the Tanzania Atomic Energy Commission, instrumentation maintenance section, or service agents. A necessary prerequisite for the effectiveness of this concept is proper and adequate training. It is understandable that no matter how highly skilled an engineer may be, he will not be competent to carry out major repairs on a variety of complex equipment unless he has had training, preferably at the manufacturer's training center. Training of a number of technicians and engineers to attain the required levels of skills is assumed.

As a result of this survey, a report was sent to the relevant government authorities, who decided to start a project to rehabilitate and improve radiological services in district and regional hospitals in mainland Tanzania. The major objectives of the project are to rehabilitate the existing serviceable X‐ray machines and to supply to districts new X‐ray machines that are easy to install, easy to operate, and easy to maintain and service. Another objective is to develop and train maintenance staff for X‐ray facilities at district hospitals. Installation of new X‐ray machines was accompanied by a full support service contract by the suppliers.^(13)^

The Tanzania Atomic Energy Commission, on the other hand, has upgraded its facilities and technical staff to be able to conduct more frequent compliance radiation safety and quality control inspection visits to the center. The instrumentation maintenance section provides repair services and basic training courses on quality control and repair of diagnostic X‐ray machines. Thus, a followup survey to determine the compliance to quality control and radiation safety standards is being done in parallel in order to assess effectiveness of these improvements.
